# ESR paper on the proper use of mobile devices in radiology

**DOI:** 10.1007/s13244-017-0589-7

**Published:** 2018-03-22

**Authors:** European Society of Radiology (ESR)

**Affiliations:** European Society of Radiology (ESR), Neutorgasse 9/2, 1010 Vienna, Austria

**Keywords:** Radiology trends, Radiology appliances, Radiology standards, Equipment & supplies, Internet

## Abstract

**Abstract:**

Mobile devices (smartphones, tablets, etc.) have become key methods of communication, data access and data sharing for the population in the past decade. The technological capabilities of these devices have expanded very rapidly; for example, their in-built cameras have largely replaced conventional cameras. Their processing power is often sufficient to handle the large data sets of radiology studies and to manipulate images and studies directly on hand-held devices. Thus, they can be used to transmit and view radiology studies, often in locations remote from the source of the imaging data. They are not recommended for primary interpretation of radiology studies, but they facilitate sharing of studies for second opinions, viewing of studies and reports by clinicians at the bedside, etc. Other potential applications include remote participation in educational activity (e.g. webinars) and consultation of online educational content, e-books, journals and reference sources. Social-networking applications can be used for exchanging professional information and teaching. Users of mobile device must be aware of the vulnerabilities and dangers of their use, in particular regarding the potential for inappropriate sharing of confidential patient information, and must take appropriate steps to protect confidential data.

**Key Points:**

*• Mobile devices have revolutionized communication in the past decade, and are now ubiquitous.*

*• Mobile devices have sufficient processing power to manipulate and display large data sets of radiological images.*

*• Mobile devices allow transmission & sharing of radiologic studies for purposes of second opinions, bedside review of images, teaching, etc.*

*• Mobile devices are currently not recommended as tools for primary interpretation of radiologic studies.*

*• The use of mobile devices for image and data transmission carries risks, especially regarding confidentiality, which must be considered.*

## Introduction

Since the introduction of the Apple iPhone 10 years ago, so-called mobile devices have become ubiquitous. Among many other uses, health applications are used both by the general public (mostly as fitness tracking or for health advice) and medical doctors. There is a large number of radiology applications (or 'apps') on the market today, and many more to come. Mobile devices are well-connected to the Internet, allowing hand-held access to all information available in the Internet. Mobile devices are also the main access mode to social media, which are expected to play an increasing role in radiology.

This text aims at clarifying the potential uses of mobile devices in radiology. Three main applications can be identified: (1) use of mobile devices for viewing or interpretation of radiology examinations, (2) use of mobile devices as educational tools and (3) mobile use of social networks in the clinical context.

## Technology

Mobile devices are relatively small (i.e. mobile) computers with a graphical touch interface and mobile Internet connection. Characteristically, the capabilities of mobile devices can be extended with additional programs (‘apps’) that can be purchased or obtained for free from so-called ‘app stores’ [1]. Mobile devices are heavily used for mobile Internet access, for navigation, for social networking and for gaming. Most mobile devices have cameras replacing classical cameras.

Photographs and other images can be sent easily from one mobile device or user to another mobile device. Emerging uses for mobile devices are virtual reality and augmented reality.

The technology of mobile devices has evolved rapidly over the last decade. Today’s high-end mobile devices have high-speed processors, memory as large as 256 gigabytes, high-resolution monitors and an LTE or 4G wireless network connection. Depending on the screen size, mobile devices can be classified as smartphones (small screen), tablets (large screen) and phablets (medium screen).

Most mobile devices integrate excellent speech recognition, but this feature is not used by radiology apps at the time of writing [1].

## Radiology applications of mobile devices

### Use of mobile devices for interpretation of radiology examinations

Mobile devices offer many potential advantages for viewing radiology examinations. Today’s mobile devices are powerful enough to handle radiology examinations with large amounts of data. It is possible to load stacks of thousands of images and scroll through them on the mobile device itself. Another option is to use streaming techniques, where all the image manipulations are done on a remote server, with the mobile device displaying the results of image calculations. Potentially, it is possible to access radiology examinations with a mobile device from any remote location, provided the Internet connection is fast enough [2].

Image manipulation on a mobile device is possible by using the screen with its multi-touch interface. This is a very intuitive way to scroll through stacks of images or to do centre/window operations, but when it comes to more complex interactions with the app, user interface design is as important (and as variable) as with desktop applications. Using the fingers to command image manipulations impairs the quality of viewing both by hiding a part of the screen and by leaving traces on the screen.

### Primary interpretation: within the local picture archiving and communication system (PACS) or in the context of teleradiology

While screens of mobile devices are *not suitable for the primary interpretation of projectional radiographs or mammography*, screen resolution, luminance and pixel size of these devices are sufficient for a technically adequate display of computed tomography (CT) examinations. *Several studies have shown that interpretation of CT examinations on tablets with high-quality screens is possible* [3–5], *but this is still not recommended for primary interpretation, since there are potentially limiting factors* [1, 6]*.* The touch screen of mobile devices must be clean to ensure visibility of all relevant details, which is difficult to ensure while commanding or manipulating the device with the tips of the fingers [7]. While control of environmental light is easy to obtain with stationary screens of radiological workstations, it is impossible to ensure this control for mobile devices. This is especially relevant since mobile devices usually have glaring screens with reflections more prominent than with workstation screens. Thus, it is only under optimal conditions that tablet screens are suitable for interpretation of CT examinations [8, 9].

There is an abundance of software (apps) allowing mobile devices to display and/or access radiological examinations [1]. Many of these apps (particularly the most modern ones) are called ‘zero-footprint’ viewers, meaning the viewer doesn’t need installation and (in the best case) doesn’t leave any data trace on the mobile device [10]. Some viewing applications are provided by PACS vendors, others are marketed by independent companies. Some of the viewers allow a user to access images remote from the originating institution, and thus can be used for such needs as on-call emergency services or for specialized services such as tele-stroke.

#### In the context of second opinions

Second opinion provision via mobile device viewing of imaging is very convenient. It does not have the same requirements for display quality and environmental control as primary reading. It allows access to specialist knowledge without the need for these specialists to move to a PACS workstation. Second-opinion reading can be provided in the context of an exchange between colleagues, inside specialized networks or for second-opinion reading platforms. With increasing use of social media in medicine and radiology, identification and contact with super-specialists will become much easier in the future.

#### In the context of clinical result viewing

At the time of writing, this is probably the most important application of mobile devices for the viewing of radiological examinations [11]. Mobile devices are becoming widely used by clinicians (mostly surgeons) who need to access imaging data while moving around the hospital. The typical application is the use of tablets during ward rounds where they are more practical than laptops. Images of a given patient can be displayed on the devices and shown to other colleagues or to the patient. In this context, the tablet is the replacement for the classical film that was displayed in the ward. Primary diagnostic quality is not the concern in this situation; the images are used to demonstrate the result of an examination, rather than to arrive at that result in the first place. Applications allowing access to the complete medical record of the patient, including imaging results, are emerging on the market.

### Use of mobile devices as educational tools and support to diagnosis

#### Attendance of webinars

The webinar has recently become a powerful tool for spreading knowledge. A webinar is a live PowerPoint presentation, which can be attended directly from locations remote to the presenter [12]. There are usually two possible ways of following a webinar: it can be followed directly during the live presentation, and is usually available online subsequently, either publicly or under subscription or payment. Usually, in addition to following the webinar online the participants can ask questions directly after the talk. The use of mobile devices has strongly increased the attendance at webinars.

#### Consultation of clinical cases

Mobile devices are the perfect medium to consult clinical cases from case collections, like the ESR Eurorad (www.eurorad.org), or the Radiopaedia collection (www.radiopedia.org). Case collections can be reviewed even when on the move, allowing, for instance, participation in learning activities during transportation [13].

#### Consultation of e-books

Many books for radiology and other medical disciplines today are available in electronic format, so-called e-books [14]. They are convenient to read both on classical tablets and large mobile phones (so-called phablets). The main advantage of e-books is the ability to have the equivalent of a library shelf readily available in a coat pocket. Another advantage is the possibility to display dynamic images or sequences of images in the e-book format, such as stack viewing or 3D viewing. Moreover, e-books permit fast searching of relevant information based on keywords. However, there are several potential inconveniences. Scrolling through a book for some people is still faster than ‘swiping’ through the virtual pages of an e-book. Personal note-taking in e-books is still not as convenient as scribbling on the pages of a paper book. The persistence of notes in an e-book is not guaranteed when a new edition replaces the older one—notes might just disappear. However, the tools for notes and information input are improving.

#### Educational applications

Apart from reading radiological examinations using the “DICOM Viewer”, smartphones and tablets are becoming increasingly important for radiologists attending conferences, and also in such everyday activities as answering specific questions, enhancing knowledge of particular topics, and keeping abreast of the latest technological and scientific discoveries [15]. A smartphone equipped with the proper application is the most appropriate tool for building a database of educational radiological cases. Moreover, tablets offer multimedia and interactive opportunities, which make them particularly suitable for e-learning.

Today, there exist several hundred smartphone applications for use in radiology [1].

The applications can be searched for directly on a smartphone or downloaded using iTunes for iOS devices or using play.google.com for Android devices.

Anatomy applications are among the most used, allowing one to navigate through examinations of different modalities, with structural annotations being provided.

E-learning applications allow interactive e-learning and searching for specific information. They can help to increase knowledge of a particular subject, find the best protocol for an examination or challenge one’s knowledge with a clinical case.

Database applications such as iDatabase (iOS) or MyData (Android) are applications that allow creation of a database by completing pre-established forms. One can promptly build one’s own database of radiological cases for educational purposes. These are easy to use and powerful search engines. Being simple text-based applications, they do not store any images.

Applications can be also used to follow journal publications or a congress. Most journal applications allow a user to check the summary of recent issues and read articles of interest. They can be used as a powerful and practical way of following the latest scientific developments. Conference applications are used in numerous congresses and are regularly (usually yearly) updated. They allow one to check the timetables of sessions, add particular items to the agenda, find one’s way to the session, find an exhibitor, or get regular alerts about ongoing events.

### Social networks access through mobile devices during clinical activity: pros and cons

Social services, such Facebook, Twitter, LinkedIn, Google Plus, and many others, have played an important role in everybody’s personal and professional life for a number of years.

Social media can be used by radiologists for the purpose of exchanging professional information with each other, visibility, social communication, networking, and searching for a job. Moreover, the users can get the latest information concerning their profession, congresses, and other events, and can use the media for e-learning and scientific monitoring.

Twitter is a microblogging social network, where users can post short messages of 140 characters maximum (increased to 280 characters on a trial basis in October 2017). These messages can contain photos or links. In this way, each user can share his interests, findings, or opinions.

In Facebook, the information can be shared by a page (personal page, official page, or by a group) which can be either open or closed (in a closed group, the administrator or one of the current group members has to accept you into the group). To get updates from a page you like or to follow it, or get an update from a group, one must be its member.

These platforms also offer great interaction opportunities, for whichever growing engagement of the users is essential. However, the identity of the persons who share the information and the source of that information cannot always be verified.

#### As a teaching tool

The benefits of social media are the access to a large user base, the potential to reach a large audience, the ease of access, the availability at point of need, and the attraction of the media for younger users [16].

Disadvantages are the lack of trusted content providers, the absence of exhaustive information, the specific limitations imposed by each social media platform, the possible difficulties of finding the needed information that can become time consuming, and privacy issues [17].

Social media can be used for educational purposes in order to follow news, to keep abreast of learning trends and developments, to promote and share interesting posts and media, and to create educational content.

However, while creating the content, some points should be kept in mind concerning security and confidentiality: one should ask oneself who is the owner of the images, if patient consent is needed, and if the images are correctly anonymized.

#### As a second opinion tool

Social networks are often used in order to get a second opinion. The second opinion is often provided inside professional groups of social networks such as Facebook or in smaller communities on WhatsApp. However, before asking for a second opinion on social networks, one should again be certain about confidentiality issues. Images should be correctly anonymized and one should have the right to share them.

## Security/legal aspects

As with all computers, mobile devices are vulnerable and special care must be taken to ensure that sensitive data stored on or communicated by these devices are protected. While general principles of computer security must be applied, there are some specific risks associated with the mobility and wireless communication that mobile devices rely on [18].

### Access protection

Every mobile device should have access protection to ensure data stored on the device and access to servers the device is connected to are protected in case a third party gets physical access to the device (i.e. loss or theft). There are several means of access protection, the most basic being a passcode. The most popular at the time of writing is probably the fingerprint, with other biometric unlocking methods emerging (e.g. face recognition, iris scanning). It is important to use one of these locking mechanisms and to be aware of its limitations.

### Remote control of the mobile device

Mobile devices used for medical applications should be configured to allow remote location/tracking of the device. In case the device cannot be recovered, remote erasure of all data stored on the device ensures no patient data can be accessed by unauthorized persons. Remote control and erasure usually must be enabled explicitly.

### Protection from malware

Mobile devices are less subject to virus/malware attacks than desktop computers. However, the number of attacks on mobile devices is increasing. The iOS platform is considered to be less vulnerable than Android-based mobile devices since (except when jailbreaking) iOS apps come only from one source, while there are several sources for Android apps.

### Wireless security

All mobile devices rely on wireless communication which is particularly vulnerable to all kind of attacks, regardless which kind of network is used (Wi-Fi, cellular). While cellular networks are encrypted by default, the encryption of Wi-Fi must be explicitly activated. Different encryption protocols can be used, with Wired Equivalent Privacy (WEP) being the oldest and least safe and Wi-Fi Protected Access 2 (WPA2) being the most recent and secure encryption protocol.

## Summary

In summary, mobile devices are largely used by the radiology community not only for viewing or for interpretation of radiology examinations, but also as educational tools and for creating social networks in the clinical and educational context. Appropriate use of mobile devices in radiology has the potential to increase awareness of the value contributed to patient care by radiology activity; given the current focus of value-based healthcare, this is an important opportunity [19]. While their use is extremely practical and offers numerous opportunities, one should work on optimizing use according to specific needs and always keep in mind possible security and confidentiality issues.
